# Experimental Study on Humidification Coagulation and Removal of Fine Particles Using an Electrostatic Precipitator

**DOI:** 10.3390/polym15092065

**Published:** 2023-04-26

**Authors:** Lichun Xiao, Xiaoyu Zhai, Yingying Han, Hongrui Chen, Hengtian Li

**Affiliations:** Hebei Key Laboratory of Heavy Metal Deep-Remediation in Water and Resource Reuse, School of Environmental and Chemical Engineering, Yanshan University, Qinhuangdao 066004, China; xyzhai0720@163.com (X.Z.); hany202210@163.com (Y.H.); chrwz6@163.com (H.C.); lihengtian@stumail.ysu.edu.cn (H.L.)

**Keywords:** wet electrostatic precipitator, fine particles, humidification coagulation, chemical coagulation, dust removal efficiency

## Abstract

A wet electrostatic precipitator (WESP) has much higher capture rate for fine particulate matter, PM_2.5_, than a traditional dry type electrostatic precipitator does. In order to make full use of existing dust removal equipment and reduce the emissions of smoke and dust to zero, a combination of chemical coagulation and humidification coagulation is proposed using a WESP. The results show that the addition of chemical coagulant can promote the coagulation of coal-fired dust particles. After the addition of pectin (PG), the median diameter of dust particles increases from 28.19 μm to 45.28 μm. Water vapor humidification can promote the coagulation of dust particles. When the water vapor injection rate increases from 0 kg/h to 3.2 kg/h, the median diameter of dust particles increases from 28.19 μm to 36.45 μm. The synergistic effect of the coagulant and water vapor can enhance the chemical coagulation effect; when 1.0 × 10^−2^ g/L PG and 3.2 kg/h water vapor synergize, the collection efficiency reaches 98.17%, and when 1.0 × 10^−2^ g/L polyacrylamide (PAM) and 3.2 kg/h water vapor synergize, the collection efficiency reaches 96.68%. Both chemical coagulation and water vapor humidification can promote the condensation of coal dust, which is beneficial to improve the efficient capture of fine particles using WESP.

## 1. Introduction

China is the largest coal producer and consumer in the world. The large amount of fine particles emitted by coal combustion is the main cause of haze weather and the reduction in environmental visibility. Fine particles suspended in the atmosphere will cause direct harm to human health. Therefore, reducing the emission of fine particles after coal combustion has become an important means for the air pollution control. At present, many places in China require coal-fired boilers to meet the ultra-low emission standard of air pollution after dust purification [1,2,3]. In 2018, the Emission Standard of Air Pollutants for Coal-fired Power Plants (DB33/2147-2018) in Zhejiang Province required that the emission limit of particulate matter generated by coal-fired power generation boilers should be less than 5 mg/m^3^ [4]. Nevertheless, according to the dust removal mechanism, if the dust particle size is less than 1 μm, it is difficult to capture because of its Brownian motion. Chemical coagulation is an economic and effective method to promote the collection of fine particle dust using an electrostatic precipitator because it can increase the particle size and turn it to large particle dust. Most of the chemical coagulants are macromolecular polymers, which is atomized in the electrostatic precipitator with the nozzle to form small droplets. Saturated water vapor is easy to generate phase change and condensation on the surface of dust, to form small droplets with fine particles as the core. Humidification and chemical coagulation are conducive to the increase in the size of fine particles, which can improve the collection efficiency of the electrostatic precipitator. Pectin (PG) is a natural organic polymer flocculant with a great flocculation effect. It has abundant sources, can be biodegradable in water, and causes no harm to human health. Polyamide (PAM) is a high-quality non-ionic polymer flocculant with a strong bridging ability for colloids, and the particles become suspended in water, and it causes low levels of environmental toxicity. Sodium carboxymethyl cellulose (CMC) is a flocculant with good chemical stability, and it is not easy to corrode and deteriorate. It is harmless to the human body. Polyaluminum chloride (PAC) is a commonly used aluminum-based inorganic polymer coagulant that quickly forms flocs in water. PAC can provide high-valent polymeric ions when it is added to water. Firstly, the particle size distribution of the original coal dust was analyzed in the study. as Additionally, microscopic photos taken after adding four types chemical agglomerating agent and performing humidification are presented. Secondly, chemical coagulation experiments were carried out in the wet electrostatic precipitator, and the influence of the coagulant type, humidification, and chemical coagulation on dust particles’ size distribution was analyzed. Finally, the influence of the chemical coagulant type, humidification, the concentration of coagulant, and other factors on the dust removal efficiency was studied via the electrostatic precipitator performance experiment. The humidification–atomization synergistic effect on chemical coagulation for fine particle dust removal is discussed too. Chemical coagulation can improve the removal efficiency of fine particles and reduce the water consumption of a wet electrostatic precipitator.

In 1995, Linak et al. [5] studied the coagulation effect of a coagulant on harmful metals. In 2002, Durham et al. [6] greatly improved the removal efficiency of an electrostatic precipitator for particulate matter by using a coagulant composed of various substances, and the stronger the polarity of the coagulant was, the better the coagulation effect was. Hogg C R [7] added surfactants to the dedusting spray system to enhance the wettability of dust; it agglomerated the fine particles and improved the dedusting efficiency. In 2003, Baldrey K E et al. [8] developed a chemical coagulant to promote the removal efficiency of an electrostatic precipitator for particles. Zhang J Yet al. [9,10,11,12,13,14] proposed that a chemical coagulant could promote an increase in the particle size, and they proved that it could significantly improve the dust removal efficiency in experiments. In 2007, Rajniak P et al. [15] discussed the effect of coagulants with different physical properties in the process of fine particle coagulation and determined the relationship between fine particles and the concentration of coagulant. In 2009, Yang L J et al. [16,17] analyzed the principle of steam phase change to promote the coagulation and growth of PM_2.5_ and proposed the feasibility of using them to remove PM_2.5_ in the wet desulfurization system. In 2011, Forbes E et al. [18] determined the trend of the influence of temperature on the coagulation effect in a humidification and coagulation experiment on the influence of chemical coagulant droplets on fine particles. In 2015, Balakin B V et al. [19] studied the effect of the capillary liquid bridge number and wetting angle on the particle coagulation efficiency based on a computational fluid dynamics model. Balakin B V and Sasutinova G et al. [20] produced the coagulation model of a coagulant solution and particles and found that the coagulation efficiency decreased significantly with the increase in particle size. In 2018, Hu Bin et al. [21,22] added steam and a chemical coagulant into desulfurization flue gas to promote its coagulation and reduce the emission of fine particles. They used a combination of chemical and turbulent coagulation in front of the electrostatic precipitator to improve the removal efficiency by PM_2.5_. Jaworek A [23] used a two-stage electrostatic precipitator in the experiment. In the first stage a charged agglomeration device was used, which resulted in coagulation of PM_2.5_. It improved the collection efficiency of fine particles. Sadighzadeh A et al. [24] added water vapor into an acoustic coagulation chamber, and via its synergistic effect, they improved the coagulation efficiency of sulfuric acid smoke. In 2019, Zhou L [25] used two polymer solutions with different chemical properties to conduct a chemical coagulation experiment, which proved that the atomization performance of a chemical coagulation solution has an important impact on the removal of fine particles. In 2020, Li RH et al. [26] sprayed chemical coagulant kappa carrageenan/Tween-80/NH_4_Cl (KC/TW/NH_4_Cl) into a turbulent coagulation chamber, and the particle diameter increased from 2.8 μm to 10.0 μm. In 2021, Zhang Z et al. [27] achieved the coagulation of dust of less than 0.5 mm with the chemical coagulation method by humidifying and injecting soap during coking coal loading and unloading. In 2022, Zhou L et al. [28] added sesbania gum (SBG) and styrene butadiene lotion (SBE) to an electrostatic precipitator, and the total dust removal efficiency increased from 89.45% to 91.05%. Respectively, with the addition of surfactant (T-100), the removal rate increased from 93.58% to 94.42%. It can be seen from previous research results that humidification and chemical coagulation can make fine particle dust thicker and larger and improve the dust removal efficiency. However, in the above studies, chemical coagulant was generally sprayed into the dry dust collector or flue gas. The liquid–gas ratio of chemical coagulant to flue gas was relatively small, the probability of collision between coagulant and fine particle dust was low, and the coagulation effect was poor. This paper proposes that a wet electrostatic precipitator can greatly improve the chemical coagulation effect by humidification and chemical coagulation, and a combination of the two methods can improve the collection performance of fine particle dust, it will provide ideas for achieving near-zero emission of atmospheric particles.

## 2. Experimental Principle and Device

### 2.1. Experimental Facility

**Figure 1:**
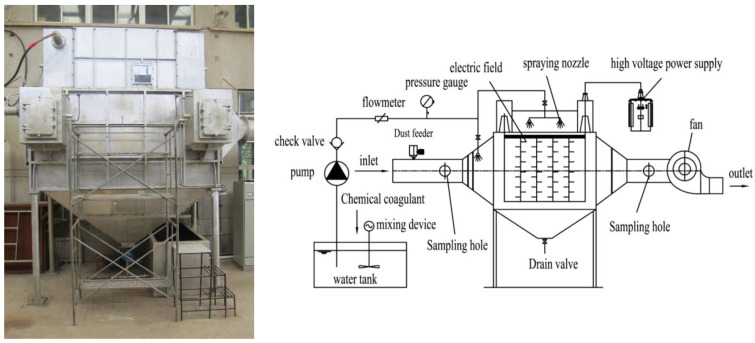
Wet electrostatic precipitator.

**Figure 2:**
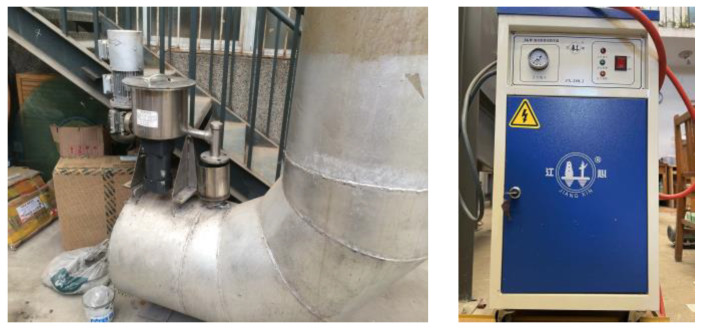
Dust emission device and water vapor generator.

The wet electrostatic precipitator system used in the experiment is shown in Figure 1. It is mainly composed of a DC high-voltage power supply system, a low-voltage control system, a fan system, a feeding system, and a spray system. The spray system is an important part of the WESP. The structural parameters of WESP are shown in Table 1. The screw feeder is an important part of the dust dosing system, as shown in Figure 2. The screw feeder is equipped with a feeding rod and a mixing blade. During the experiment, the frequency of the frequency converter was adjusted to make the powder fall into the inlet pipe at a certain speed. Under the effect of the gas flow field, air and dust quickly mix to form a dusty gas and enter the wet electrostatic precipitator. The water vapor generator is shown in Figure 2. It is composed of an electric heater, a pressure controller, a pressure gauge, a water tank, a scale gauge, an air outlet valve, a safety valve, a water supply valve, and a drain valve.

### 2.2. Measuring Technique

Before the precipitator experiment, a coagulant solution at a preset concentration in the circulating water tank was prepared, and a dust collection container in the ash hopper was placed at the bottom of the electrostatic precipitator. During the experiment, a feeder was used to emit dust, and a steam boiler was used to add water vapor. At the same time, the dust concentration at the inlet and outlet of the electrostatic precipitator was measured. After the experiment, the dust collection container was removed from the ash hopper, and the mixed liquid collected in the container was left to stand for 20 to 30 min. After the dust had completely settled, the waste water was discarded, the dust was collected in a container, the BT-9300H laser particle size distribution instrument produced by Dandong Baite Instrument Co., Ltd. was used to measure the particle size distribution of the dust. The instrument manufacturer is located in Dandong City, Liaoning Province, China.The working principle is to use a sample preparation device to transport the sample to the measurement area. After reaching the measurement area, the laser irradiated the sample and generated a specific light scattering signal. These light scattering signals were converted into electrical signals by a photodetector inside the instrument, which were processed specifically by a computer to obtain particle size distribution results. The model of scanning electron microscope is KYKY-2800B. Its manufacturer is Scientific Instrument Factory of Chinese Academy of Sciences. For the dust content measurement, the isokinetic sampling method was adopted.

**Figure 3:**
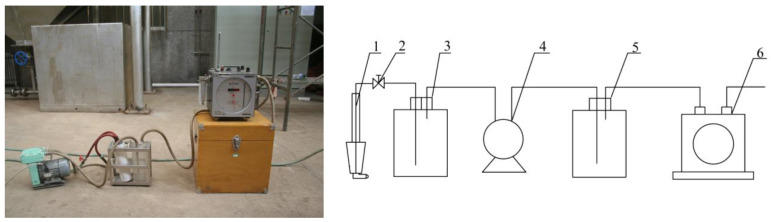
Dust density measuring device. 1. Sampling pipe. 2. Regulating valve. 3. Drying bottle. 4. Vacuum pumping pump. 5. Buffer bottle. 6. Flowmeter.

The sampling system is shown in Figure 3. During the experiment, the flow rate in the sampling pipe and the flue gas in the duct should be as similar as possible, so that the results obtained are more accurate. The sampling operation process is as follows: The sampling pipe was connected to a drying bottle, an air extraction pump, a buffer bottle, and a flowmeter in turn, and the sampling pipe with a filter tube was installed at the sampling point. The flow rate of the flowmeter was adjusted when sampling. Written down the flowmeter before and after sampling. The ratio of the filter tube weight difference to the volume flow was dust content.

### 2.3. Experimental Materials

The source of the dust is from Qinhuangdao Power Generation Co., Ltd. (Qinhuangdao, China). The factory is located in Qinhuangdao City, Hebei Province, China.The experimental materials is shown in Table 2.

## 3. Results and Discussion

### 3.1. Particle Size Distribution of Coal Dust

**Figure 4:**
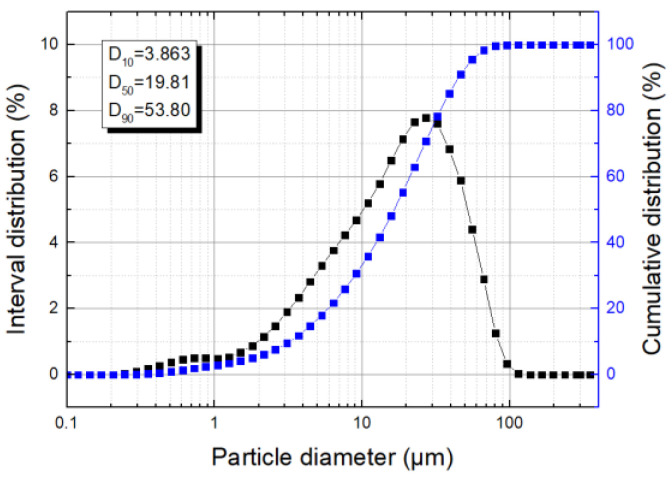
Particle size distribution of coal dust.

In the experiment, the BT-9300H laser particle size analyzer was used to measure the particle size distribution of coal dust. The particle diameter is represented by the volume distribution. D_10_, D_50_, and D_90_ are the particle sizes corresponding to the dust content at 10%, 50%, and 90%, respectively. The results are shown in Figure 4.

It can be seen that the particle size of coal dust is relatively small, D_50_ = 19.81 μm. About 10% of the dust particles are less than 3.863 μm, about 50% of the dust particles are less than 19.81 μm, and about 90% of the dust particle sizes are than 53.80 μm. Among them, 13.62% have particle sizes below 5 μm, and 28.2% have particle sizes below 10 μm. These particles can enter the upper respiratory tract of the human body and stay in ambient air for a long time, which is very harmful to human health and atmospheric visibility.

### 3.2. Morphology Analysis of Coal Dust

**Figure 5:**
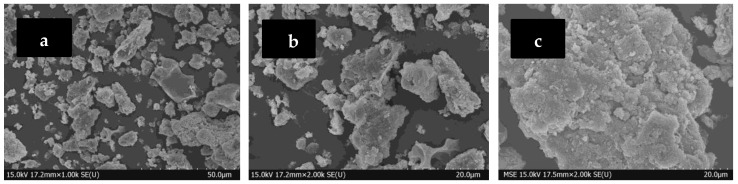

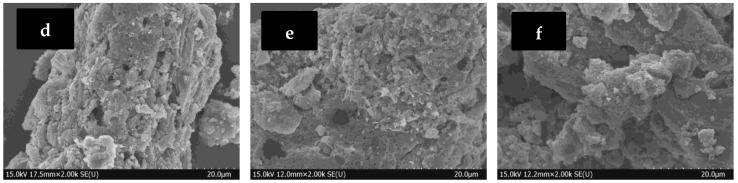
Microscopic morphology of coal dust. (**a**,**b**) Original dust. (**c**) Dust added with PG. (**d**) Dust added with PAM. (**e**) Dust added with PG and water vapor. (**f**) Dust added with PAM and water vapor.

The morphology of the dust was analyzed via scanning electron microscopy, and the amplified morphology of the dust surface before and after the chemical coagulation experiment was obtained, as shown in Figure 5. It can be seen that the original dust particles have various shapes, generally presenting irregular blocky shapes and loose textures. Some particles also combined with other particles to form larger particle clusters. The reason is that dust is generated during the combustion process and is accompanied by many chemical changes. During the cooling process, combustion products combine and condense together to form particle clusters of different sizes. After injecting PG, PAM, and water vapor into the electrostatic precipitator, the dust particles significantly increased, and small particles with different particle sizes combined with larger particles to form larger aggregates. In Figure 5c, the middle floc has a large block shape, and the dust closely connected. In Figure 5d, although the middle floc is large, there are many holes on the scanning surface and the connections are loose. In Figure 5e,f, after adding water vapor in the ESP, the size of the coagulant particles further increase, and under the synergistic action of the coagulant and water vapor, more small particles adhere to the long network chain of the coagulant.

### 3.3. Influence of Humidification on Dust Coagulation

#### 3.3.1. Influence of Coagulant Types on Dust Coagulation

**Figure 6:**
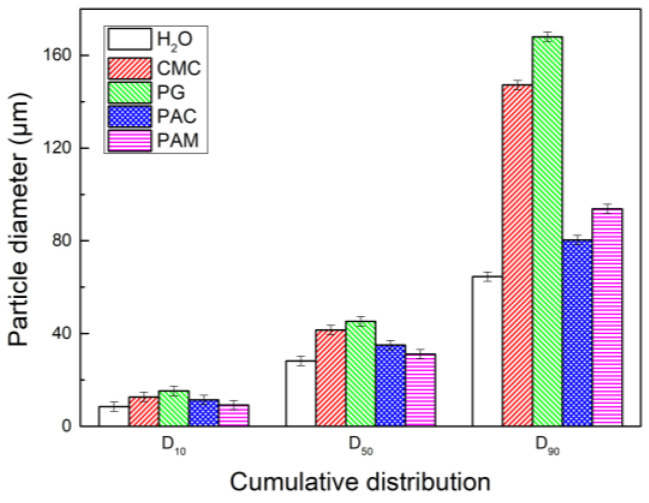
Histogram of cumulative particle size distribution of coagulant types.

The type of chemical coagulant is an important factor affecting the coagulation and growth of fine particles. Four coagulants, CMC, PG, PAC, and PAM, were used in this paper. The concentration was adjusted to 1.0 × 10^−2^ g/L, and it was successively added to the circulating water tank; the adding point is shown in Figure 1. The results are shown in Figure 6. It can be seen that there is obvious difference in the corresponding D_50_ and D_90_ particle sizes of the four coagulants. The D_90_ of PG and CMC is much larger than that of PAC, PAM, and water. The order in which D_90_ changes from large to small is PG, CMC, PAM, PAC, and then water, and the corresponding particle sizes are 168 μm, 147.2 μm, 93.78 μm, 80.33 μm, and 64.55 μm. This shows that when the coagulant solution is sprayed into the dust collector in the form of droplets, the droplets will collide with the dust particles, and the particles will adsorb the coagulant droplets, connecting two or more particles together by “bridging” to form flocs. Due to the influence of liquid viscosity and surface tension on the diameter and quantity of fog droplets, PG has a greater viscosity than CMC does, with a larger diameter of mist droplets and the best coagulation performance. The disadvantage is that a high spray pressure is required to maintain the atomization of mist droplets. In addition, PG is a natural high molecular compound, which can become a stable hydrophilic viscous colloid in case of water; it can increase the probability of trapping particles and promote the coagulation of dust particles. The main reason why PAM has a better coagulation performance than PAC does is that PAM has a certain number of polar groups, and the van der Waals force generated between PAM and particles is greater than that of PAC. The molecular chain of PAM is also much larger than that of PAC; so, the particle size of the flocs produced by PAM is also larger than that of PAC. Simply spraying water also has a certain coagulation effect, mainly due to the increased adhesion of dust.

#### 3.3.2. Influence of Water Vapor Humidification on Dust Coagulation

**Figure 7:**
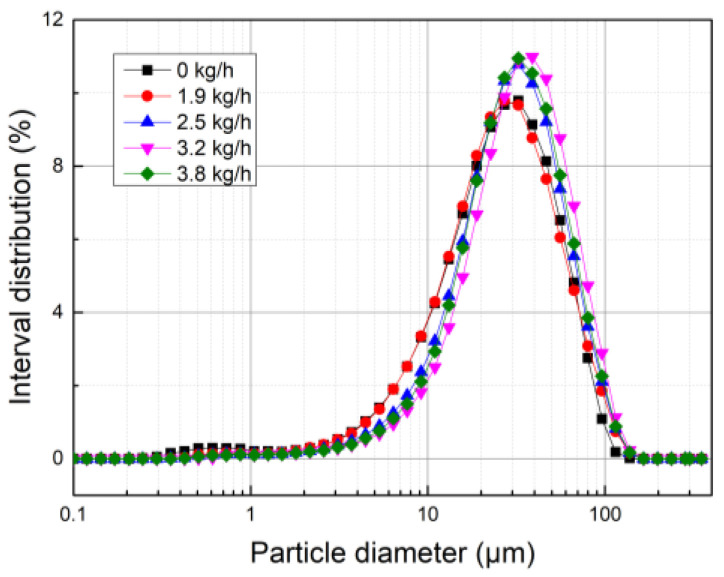
Particle size distribution diagram of dust coagulation caused by water vapor humidification.

As shown in Figure 1, by spraying water vapor mass flow at 0 kg/h, 1.9 kg/h, 2.5 kg/h, 3.2 kg/h, and 3.8 kg/h at the inlet of the WESP, the influence of water vapor humidification on dust coagulation effect in coal-fired power plant could be explored. The results are shown in Figure 7. It can be seen that after water vapor is injected, the curve moves to the right. When the water vapor injection rate is 3.2 kg/h, the curve is on the far right. When the water vapor injection rate is 0, the corresponding D_10_, D_50_, and D_90_ are 8.511 μm, 28.19 μm, and 64.55 μm. When the water vapor injection rate is 3.2 kg/h, the corresponding D_10_, D_50_, and D_90_ reach 13.43 μm, 36.45 μm, and 77.58 μm. This shows that the addition of water vapor is conducive to the coagulation of dust particles in a WESP.

**Figure 8:**
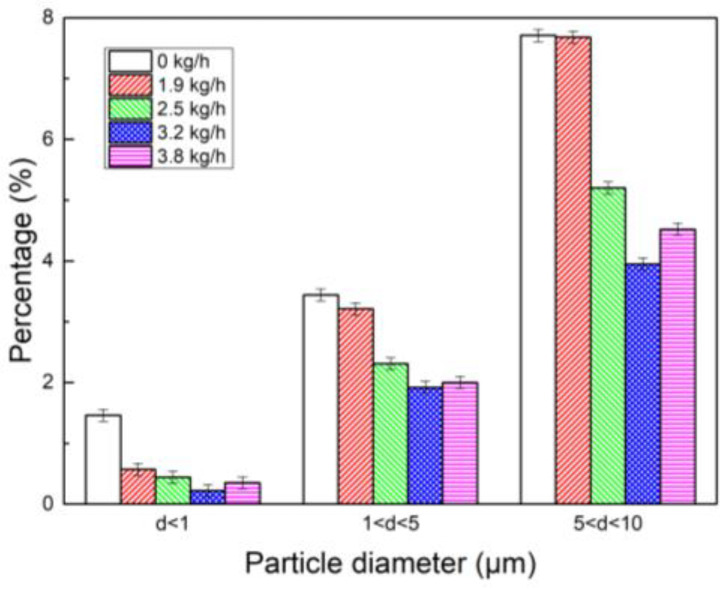
Histogram of particle size interval content of dust coagulation caused by water vapor humidification.

Figure 8 more intuitively shows the effect of water vapor injection on the content of fine dust. It can be seen that the content of fine dust in three sections, of which the particle diameters were d < 1 μm, 1 μm < d < 5 μm, and 5 μm < d < 10 μm, decreased after the injection of water vapor. When the water vapor injection rate was 3.2 kg/h, the content of particles with a particle size between 1 and 5 μm decreased from 3.44% to 1.92%, and the content of particles with particle size between 5 and 10 μm decreased from 7.71% to 3.95%. This also proves that spraying water vapor is conducive to dust coagulation. This is because by injecting water vapor, molecules will quickly adsorb on the surface of the particles as condensation nodules and form tiny liquid droplets due to the low temperature of the flue gas. It will increase the water content of the particles and enhance the adhesion of dust particles themselves. At the same time, after water vapor has been injected, the moisture content in the flue gas will increase. At this time, water vapor molecules will form a film on the dust surface, which can improve the conductivity of dust, reduce the surface specific resistance of dust particles, and make the specific resistance fall within the appropriate dust collection range. Due to the use of a certain concentration of particulate matter, there is also a limit to the number of condensation nuclei generated when water vapor is injected. Therefore, when the injection amount is greater than 3.8 kg/h, the number of droplets containing dust generated by condensation does not increase, and the coagulation effect does not increase.

#### 3.3.3. Synergistic Effect of Water Vapor and PG on Dust Coagulation

**Figure 9:**
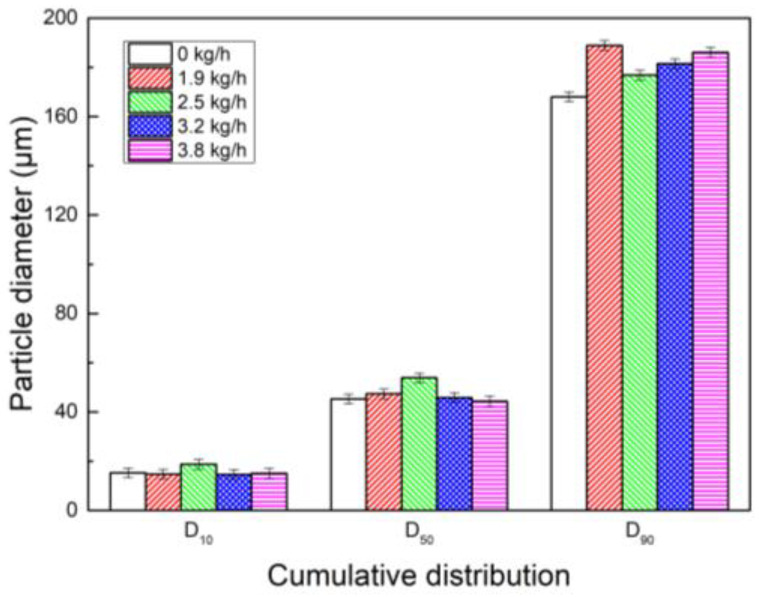
Histogram of cumulative particle size distribution of synergistic effect of PG and water vapor.

When the coagulant PG’s content is 1.0 × 10^−2^ g/L, by successively spraying water vapor mass flow at 0 kg/h, 1.9 kg/h, 2.5 kg/h, 3.2 kg/h, and 3.8 kg/h in combination with PG, the synergistic effects on dust coagulation were studied. The results are shown in Figure 9. It can be seen that the particle size of dust increased after PG was used in combination with water vapor. When only PG was added, the D_10_, D_50_, and D_90_ of the corresponding dust particles were 15.26 μm, 45.28 μm, and 168 μm. When water vapor mass flow was sprayed at 2.5 kg/h and PG was sprayed at the same time, D_10_ and D_50_ rose to 18.75 μm and 53.86 μm, respectively. When water vapor mass flow was sprayed at 1.9 kg/h and PG was sprayed at the same time, D_90_ rose to 188.9 μm. This shows that the synergistic effect of water vapor and PG is better than the coagulation effect of PG. Water vapor and PG are conducive to the coarsening and growth of dust particles.

**Figure 10:**
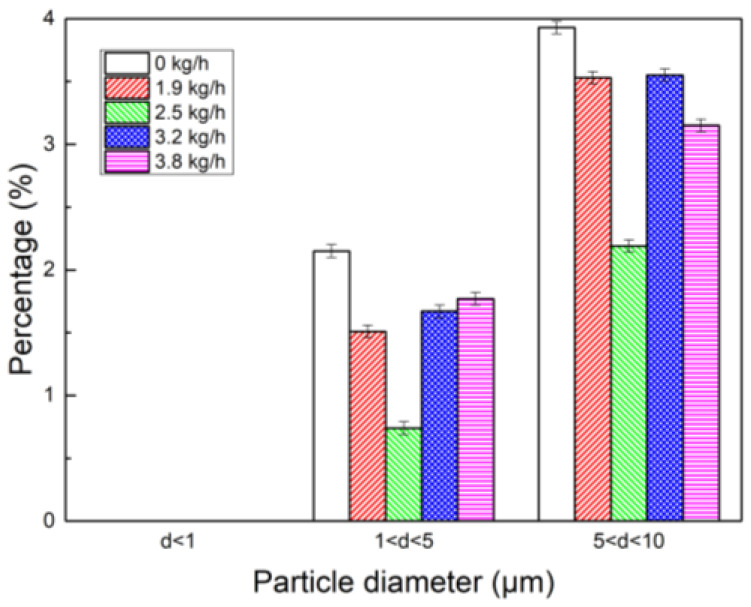
Histogram of particle size interval content of synergistic effect of PG and water vapor.

In order to see the content of fine dust more intuitively, a histogram of the dust particle size range content was drawn. It can be seen from Figure 10, compared with the addition of PG, the dust content in each particle size range was reduced to varying degrees after the combined use of water vapor and PG. When water vapor was sprayed at 2.5 kg/h and PG was sprayed at the same time, the content of particles with a particle size of 1~5 μm decreased from 2.15% to 0.74%, and the content of particles with particle size of 5~10 μm decreased from 3.93% to 2.19%. It is proved that the synergistic effect of water vapor and PG can reduce the content of fine dust. According to the theory of the hetero nucleation of water vapor on the surface of fine particles, when the water vapor is supersaturated, entropy S > 1, and when the diameter of the embryo droplet is greater than the critical radius, ΔG < 0, particles spontaneously condense and grow. When water vapor and PG were added together, the diameter of the embryo droplet increased, and therefore, the spontaneous coagulation effect of fine particles is better than if water vapor was added alone.

#### 3.3.4. Synergistic Effect of Water Vapor and PAM on Dust Coagulation

**Figure 11:**
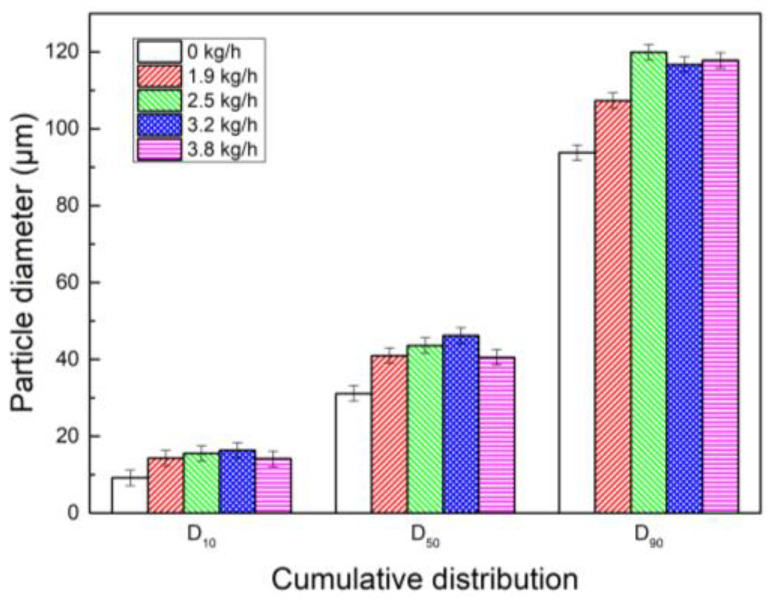
Histogram of cumulative particle size distribution of synergistic effect of PAM and water vapor.

The concentration of coagulant PAM is 1.0 × 10^−2^ g/L, and by successively spraying water vapor mass flow at 0 kg/h, 1.9 kg/h, 2.5 kg/h, 3.2 kg/h, and 3.8 kg/h in combination with PAM, their synergistic effects on dust coagulation were studied. The results are shown in Figure 11. It can be seen that the particle size of dust increased after PAM and water vapor were sprayed. When only PAM was added, the D_10_, D_50_, and D_90_ of the corresponding dust particles are 9.181 μm, 31.12 μm, and 93.78 μm. When water vapor was added at 3.2 kg/h with PAM, D_10_ and D_50_ reached 16.35 μm and 46.23 μm, respectively. When water vapor mass flow was added at 2.5 kg/h in combination with PAM, D_90_ reaches 119.9 μm. This shows that the synergistic effect of water vapor and PAM is better than the coagulation effect of PAM is, and water vapor and PAM are conducive to the coarsening and growth of dust particles.

**Figure 12:**
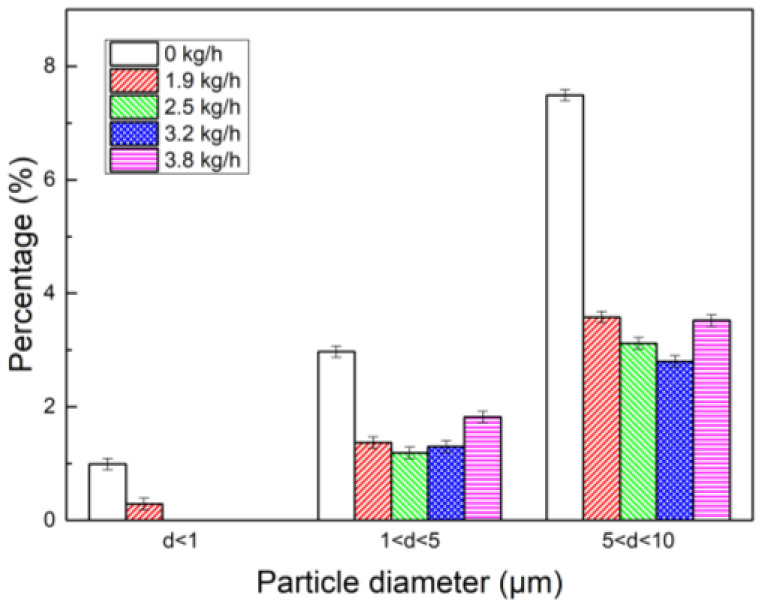
Histogram of particle size interval content of synergistic effect of PAM and water vapor.

Figure 12 shows the content of fine dust more intuitively. It can be seen that compared with the addition of PAM, the dust content in each particle size range is significantly reduced after the combination of water vapor and PAM. When water vapor was sprayed at 2.5 kg/h in combination with PAM, the content of particles with a particle size of 1~5 μm decreased from 2.97% to 1.19%. When water vapor was sprayed at 3.2 kg/h in combination with PAM, the content of particles with particle size of 5~10 μm decreased from 7.49% to 2.8%, that is, the synergistic effect of water vapor and PAM can effectively reduce the content of fine dust. It can be seen that the synergistic effect of water vapor and the coagulant can enhance the effect of chemical coagulation, which is conducive to the coarsening and growth of dust particles, and the synergistic effect can effectively reduce the content of fine dust. This is because the smaller the particle size is, the larger the specific surface area of the particles is, the stronger the adsorption of water vapor is, and the easier it is to form a liquid bridge between the particles. The formation of a liquid bridge increases the force between the particles. Therefore, more fine particles gather together, and this reduces the content of fine dust and enhances the coagulation effect of subsequent chemical coagulation. According to the mechanism of changing the surface characteristics of fine particles and the bridging mechanism of polymer coagulants, PG has a more obvious surface structure and wettability on fine particle dust. Therefore, PG has a better coagulation effect than PAM does, and its dust D_50_ and D_90_ after coagulation are greater than the latter ones are.

### 3.4. Influence of Water Vapor Humidification and Coagulation on Dust Removal Efficiency

In order to further investigate the effect of chemical coagulant and water vapor on the treatment of coal-fired dust in WESP, the dust concentration at the inlet and outlet on different conditions was tested, and the dust removal efficiency was calculated.

#### 3.4.1. Influence of Coagulant Type on Dust Removal Efficiency

**Figure 13:**
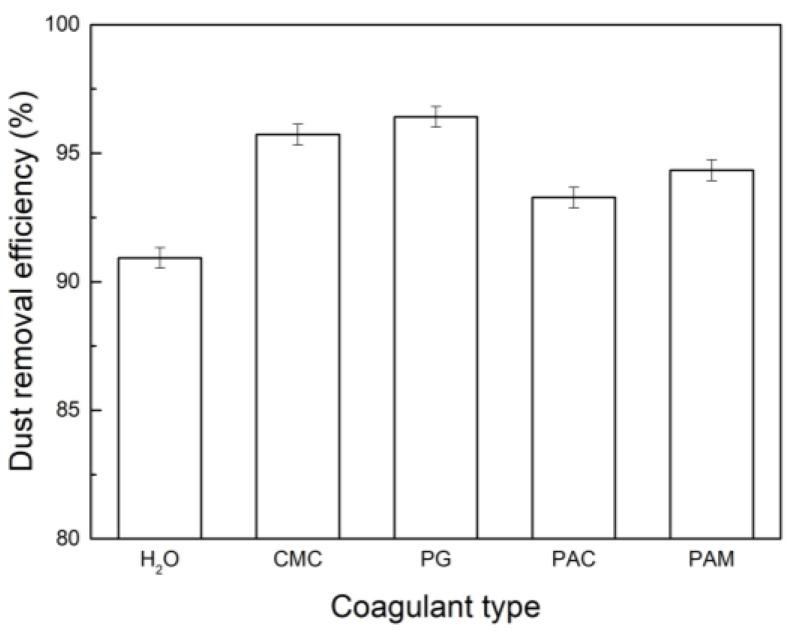
Influence of coagulant type on dust removal efficiency.

Different chemical coagulants have different coagulation effects on dust, which affect the removal efficiency of dust. The working voltage of WESP is 40 kV, and the concentration of coagulant is 1.0 × 10^−2^ g/L; the results are shown in Figure 13. It can be seen that compared with water, the dust removal efficiency was improved after adding the coagulant. For coal dust, when the coagulant was PG, the dust removal efficiency was the highest: 96.42%. When the coagulant was CMC, the dust removal efficiency was 95.73%. It can be seen that after adding PG, due to the reduction in the content of fine particles of dust, the dust removal efficiency was improved compared to that when just water was sprayed. Among the four coagulants, PG, CMC, PAM, and PAC, PG has the best coagulation effect, which coincides with the highest dust removal efficiency. This further demonstrates that chemical coagulation can effectively improve the dust removal efficiency.

#### 3.4.2. Synergistic Effect of Water Vapor and PG on Dust Removal Efficiency

**Figure 14:**
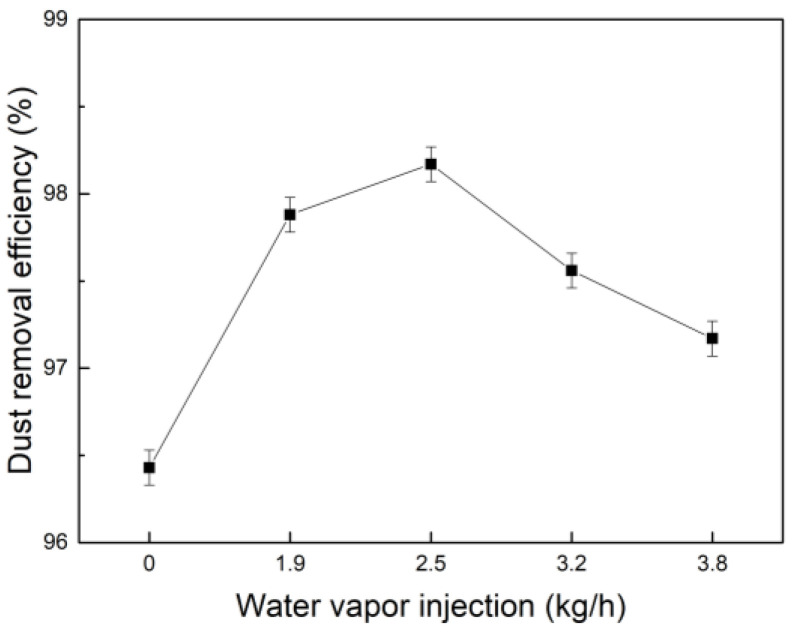
Dust removal efficiency diagram of synergistic effect of water vapor and PG.

The concentration of coagulant PG is 1.0 × 10^−2^ g/L, which was combined with water vapor mass flow sprayed at 0 kg/h, 1.9 kg/h, 2.5 kg/h, 3.2 kg/h, and 3.8 kg/h; the results are shown in Figure 14. It can be seen that the dust removal efficiency increased when water vapor and PG were sprayed together. When water vapor mass flow was added at 2.5 kg/h and PG was added at the same time, the dust removal efficiency was the largest, about 98.17%. It can be seen that by using action the same coagulant, when the rate of water vapor injected is high, the dust removal efficiency decreases instead. The reason is that due to the limitation of the concentration of fine particle dust, there is a limit to the number of fine particle dust and droplet collisions. When the rate of water vapor injected is high, there is also a maximum value for the amount of condensation nucleation and droplet formation. Therefore, the change of dust removal efficiency is consistent with the change of D_50_ in Figure 9.

#### 3.4.3. Synergistic Effect of Water Vapor and PAM on Dust Removal Efficiency

**Figure 15:**
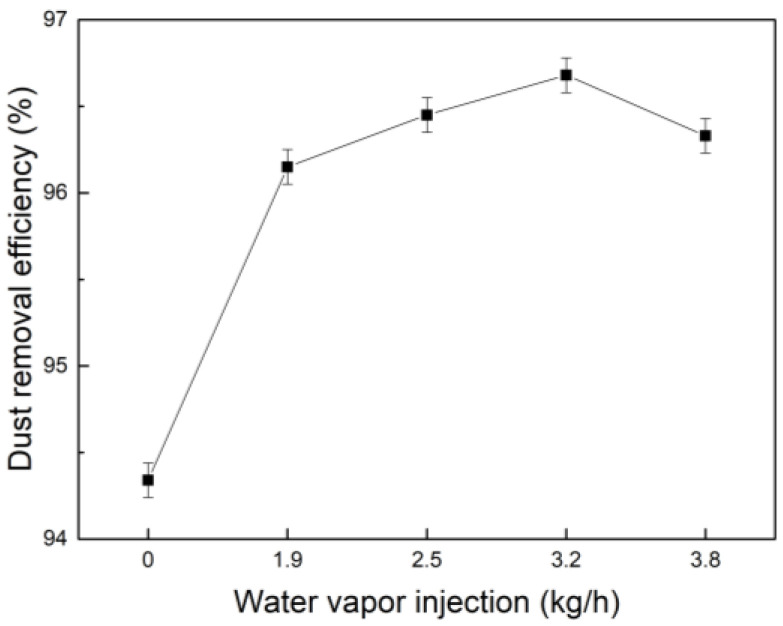
Dust removal efficiency diagram of synergistic effect of water vapor and PAM.

The concentration of coagulant PAM was 1.0 × 10^−2^ g/L, which was used in combination with water vapor mass flow sprayed at 0 kg/h, 1.9 kg/h, 2.5 kg/h, 3.2 kg/h, and 3.8 kg/h; the results are shown in Figure 15. It can be seen that after spraying water vapor, the dust removal efficiency of PAM and water vapor is significantly higher than that when only PAM was used. When only PAM was added, the mass concentration at the outlet of the dust collector is 8.48 mg/m^3^, and the dust removal efficiency is 94.34%. With the increase in water vapor content, the dust removal efficiency began to increase. When PAM was used and water vapor was sprayed at 3.2 kg/h at the same time, the mass concentration at the outlet of the dust collector decreased to 6.48 mg/m^3^, and the dust removal efficiency increased to 96.68%. However, when the water vapor rate is higher than 3.2 kg/h, it continues to increase it, and the dust removal efficiency does not increase; it declines. Comparing Figure 14 with Figure 15, it can be seen that due to the poor coalescence efficiency of PAM compared to that of PG under the same conditions, PAM requires more water vapor to condense into nuclei to form dust coalescence; so, the amount of water vapor consumed to improve the dust removal efficiency is greater than that of PG.

## 4. Conclusions

In the coal dust coagulation experiment and dust removal efficiency experiment, the following conclusions were obtained.

1. The addition of a chemical coagulant can promote the removal of fine particles from coal combustion. After adding the coagulant, the median diameter of dust increases, and large particles increase. The coagulation effects of the four coagulants used in the experiment are PG, CMC, PAM, and PAC from the strongest to weakest, and the corresponding removal efficiencies are 96.42%, 95.73%, 94.34%, and 93.28%, respectively.
2. Spraying water vapor for humidification can promote the coagulation and thickening of dust. When the injection rate of water vapor is 0 kg/h, the median diameter of coal-fired dust is 28.19 μm. When the water vapor injection rate is 3.2 kg/h, the median diameter of coal-fired dust is 36.45 μm.
3. The synergistic effect of chemical coagulant and water vapor can enhance the coagulation effect. When 2.5 kg/h water vapor and 1.0 × 10^−2^ g/L PG synergize, the removal efficiency of coal-fired dust can reach 98.17%. When 3.2 kg/h water vapor and 1.0 × 10^−2^ g/L PAM synergize, the removal efficiency of coal-fired dust can reach 96.68%.

In conclusion, when a WESP is used to treat coal dust, humidification coagulation can promote the condensation of dust particles and effectively improve the dust removal efficiency of the WESP.

## Figures and Tables

**Table 1:** Experimental Parameters of WESP.

Name	Experimental Parameters
Gas flow (m^3^/h)	10,000
Water pump motor power (kW)	7.5
Fan motor power (kW)	15
Working voltage of ESP (kV)	45
Polar matching	BS barbed wire, 480C
Electric field wind speed (m/s)	1.1
Number of electric field channels	4
Relative humidity of air (%)	20~60
Dust collector size (length × width × height) (m)	2.8 × 1.4 × 5.8
Duct diameter (mm)	500
Gas temperature (°C)	18~25
Inlet dust content (mg/m^3^)	120

**Table 2:** Experimental materials.

Reagent Name	Molecular Weight	Characteristics	Specifications	Manufacturer
Pectin (PG)	1 × 10^4^~4 × 10^5^ [29]	natural organic polysaccharide	Analytically pure	Inner MongoliaFufeng Biotechnology Co., Ltd. Zhalantun City, Inner Mongolia, China
Non ionic polyacrylamide (PAM)	1.2 × 10^7^~1.5 × 10^7^ [30]	Organic polysaccharide	Analytically pure	Tianjin Kemio Chemical Reagent Co., Ltd. Tianjin, China
Sodium carboxymethyl cellulose (CMC)	2.42 × 10^6^ [31]	natural organic polysaccharide	Analytically pure	Tianjin Kemio Chemical Reagent Co., Ltd. Tianjin, China
Polyaluminum Chloride (PAC)	1500~3000 [32]	inorganic polymer flocculant	Analytically pure	Tianjin Kemio Chemical Reagent Co., Ltd. Tianjin, China

## Data Availability

Not applicable.

## Nomenclature

d	Particle diameter
D_10_	Diameter when the cumulative distribution of dust is 10%
D_50_	Diameter when the cumulative distribution of dust is 50%
D_90_	Diameter when the cumulative distribution of dust is 90%
